# Usability evaluation of mHealth apps for elderly individuals: a scoping review

**DOI:** 10.1186/s12911-022-02064-5

**Published:** 2022-12-02

**Authors:** Qiuyi Wang, Jing Liu, Lanshu Zhou, Jing Tian, Xuemei Chen, Wei Zhang, He Wang, Wanqiong Zhou, Yitian Gao

**Affiliations:** Clinical Nursing Department, Naval Medical University, 800 Xiang Yin Road, Yangpu District, Shanghai, 200433 China

**Keywords:** mHealth, Applications, Usability, Elderly, Human factor design

## Abstract

**Background:**

Usability is a key factor affecting the acceptance of mobile health applications (mHealth apps) for elderly individuals, but traditional usability evaluation methods may not be suitable for use in this population because of aging barriers. The objectives of this study were to identify, explore, and summarize the current state of the literature on the usability evaluation of mHealth apps for older adults and to incorporate these methods into the appropriate evaluation stage.

**Methods:**

Electronic searches were conducted in 10 databases. Inclusion criteria were articles focused on the usability evaluation of mHealth apps designed for older adults. The included studies were classified according to the mHealth app usability evaluation framework, and the suitability of evaluation methods for use among the elderly was analyzed.

**Results:**

Ninety-six articles met the inclusion criteria. Research activity increased steeply after 2013 (n = 92). Satisfaction (n = 74) and learnability (n = 60) were the most frequently evaluated critical measures, while memorability (n = 13) was the least evaluated. The ratios of satisfaction, learnability, operability, and understandability measures were significantly related to the different stages of evaluation (*P* < 0.05). The methods used for usability evaluation were questionnaire (n = 68), interview (n = 36), concurrent thinking aloud (n = 25), performance metrics (n = 25), behavioral observation log (n = 14), screen recording (n = 3), eye tracking (n = 1), retrospective thinking aloud (n = 1), and feedback log (n = 1). Thirty-two studies developed their own evaluation tool to assess unique design features for elderly individuals.

**Conclusion:**

In the past five years, the number of studies in the field of usability evaluation of mHealth apps for the elderly has increased rapidly. The mHealth apps are often used as an auxiliary means of self-management to help the elderly manage their wellness and disease. According to the three stages of the mHealth app usability evaluation framework, the critical measures and evaluation methods are inconsistent. Future research should focus on selecting specific critical measures relevant to aging characteristics and adapting usability evaluation methods to elderly individuals by improving traditional tools, introducing automated evaluation tools and optimizing evaluation processes.

**Supplementary Information:**

The online version contains supplementary material available at 10.1186/s12911-022-02064-5.

## Background

Socioeconomic development in most regions worldwide has been accompanied by large reductions in fertility and equally substantial increases in life expectancy, which have led to an increase in both the number and the proportion of older people [1]. The number of adults aged 65 or older worldwide is projected to grow rapidly, rising from 727 million in 2020 to 1.5 billion in 2050 [2]. As individuals age, their intrinsic capacities decline, and the risk of multimorbidity increases, resulting in the need for ongoing monitoring or treatment [3]. However, there is a disconnect between health-care needs and health-care utilization in older people who is caused by the high cost of medical expenses, the shortage of medical human resources, and the lack of access to health services due to functional constraints [4]. To breakdown the above barriers, internet-based mobile health services have emerged. Mobile health (mHealth) refers to medical and public health services supported by mobile devices, and a software platform on such devices is called a mHealth app, with an estimation number of 325,000 in 2017 [5, 6].

In 2019, the adoption rate of smartphones by older adults aged 55–91 years was 40–68% [7]. In this context, mHealth is a promising tool for promoting healthy aging through evidence-based self-management interventions that help older adults maintain functional ability and independence [8]. The effectiveness of mHealth in promoting healthy behavior and managing chronic diseases has been proven [9]. Nevertheless, the acceptance of mHealth tools by the elderly has been limited [10], with 43% seniors over 70 quit using them during the first 14 days [11]. Usability is considered a vital factor influencing the adoption of mHealth by the elderly [12, 13], which is defined as “the extent to which a system can be used by specified users to achieve specified goals with effectiveness, efficiency and satisfaction in a specified context of use” [14, 15]. Effectiveness, efficiency and satisfaction are the critical measures of usability and thus the key points of evaluation [16]. A usable mHealth app with an age-friendly interface has many benefits for elderly individuals, including enhancing their well-being, increasing accessibility and reducing the risk of harm [17–19]. At present, a number of published standards have pointed out that usability evaluation is an indispensable step in the development of mHealth apps, and call for combining through the usability evaluation methods from empirical research [20–22].

Several reviews have been conducted to identify usability methods for mHealth apps. Zapata et al. reviewed empirical usability methods for mHealth apps by analyzing 22 studies [23]. Four evaluation methods were identified: questionnaires, interviews, logs and thinking aloud. After four years, the review was updated to include 133 articles [24], suggesting that further research should explore which methods are best suited for the target users according to their physiology and health conditions [24]. Considering the particularities of the disease, Davis et al. provided a review of usability testing of mHealth interventions for HIV [25]. In summary, previous reviews have three limitations. First, usability methods suitable for older adults have not received attention. As the elderly generally face physical, cognitive, and perceptual barriers and have lower overall familiarity with technology [26], the evaluation methods they use may be different from those of other age groups. Inappropriate methods may increase the cognitive load of elderly individuals, leading to inaccurate assessment results. Second, the global mHealth app market size was valued at USD 40.05 billion in 2020, significantly higher than in 2015 [27]. It is very likely that the types of usability evaluation methods employed have been optimized or broadened. Thus, it is necessary to reinvestigate the methods currently being used. Third, user-centered design is a powerful framework for creating easy-to-use and satisfying mHealth apps, which can be divided into three phases: requirements assessment, development, and post release [28, 29]. Choosing the appropriate usability methods at different phases can improve the cost-effectiveness of development. However, clear guidance for method selection has not been provided in the existing reviews. Based on previous literature [30–33], the mHealth app usability evaluation framework (Table 1) was proposed to identify the evaluation timeline and focus of usability, including three stages.

**Table 1:** The classification criteria for the mHealth app usability evaluation framework

Stage	Classification criteria
Stage one: Combining components	The evaluation took place in a laboratory setting and involved “user-task-system” interactions with a goal of diagnosing and fixing problems; typically based on small studies [30, 31]
Stage two: Integrating system into setting	The evaluation took place in the actual environment and involved “user-task-system-environment” interactions with a goal of testing the usability of the system in the absence of researcher influence and fixing usability problems; typically based on small studies [30, 32]
Stage three: Routine use	This stage was routine use of a complete or near-complete system in a realistic condition that could be used to determine whether the design met specific measurable performance and/or satisfaction goals or to establish a usability benchmark or make comparisons [33]

Based on the above analysis, there is a need to focus on the usability evaluation process of mHealth apps for the elderly and classify the evaluation approaches according to the mHealth app usability evaluation framework. The aim of this study includes (1) identifying, exploring, and summarizing the current state of the literature on the usability evaluation of mHealth apps for older adults and (2) incorporating evaluation methods into the appropriate stages. We performed a scoping review, as our aim is to map the literature on usability testing rather than seeking to answer a specific question by looking only for the best available information.

## Methods

TO complete this scoping review, the framework developed by Arksey and O’Malley was followed [34]. The reporting of this study followed the instructions suggested by the PRISMA extension for scoping reviews (Additional file 1: Multimedia Appendix S1).

### Identifying the research question

The following research questions were established to guide this review: (1) What is the current state of the literature that addresses usability evaluation for developing mHealth apps relevant to older adults? (2) What health conditions/diseases are being addressed by the apps that employ usability evaluation? (3) What critical measures of usability are addressed in these studies? (4) What empirical methods and techniques are used to evaluate usability?

### Searching for relevant studies

**Fig. 1:**
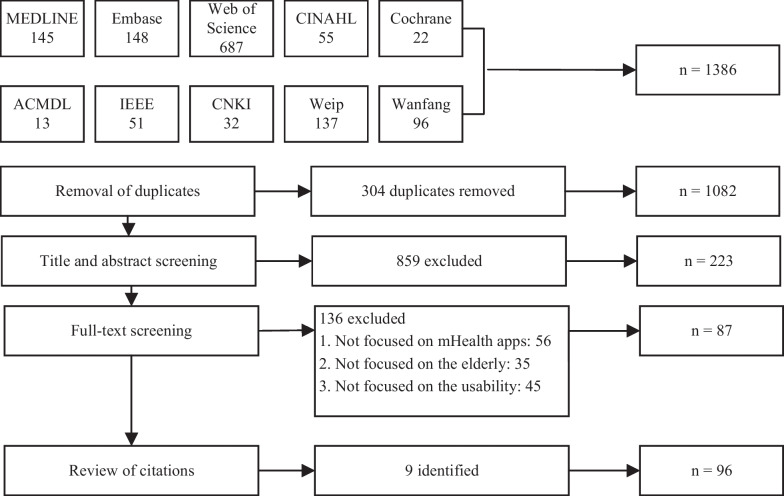
Flowchart of the study selection process

Ten databases shown in Fig. 1 of different disciplines were searched, such as medicine, nursing, allied health, computer and engineering sciences. The following keywords were identified and combined to address the research questions: (1) mobile devices, (2) the software used in the devices, (3) improving health as the main purpose, (4) mobile health, (5) usability as the research topic, and (6) the elderly as the target population. Chinese synonyms were used to maximize inclusion. Keywords and related subject headings were searched using Boolean operators. The search string is shown in Table 2. Finally, the reference lists of the included studies were reviewed to identify additional studies.

**Table 2:** Search string

Scope	String
Mobile device	(smartphone OR smart phone OR touchscreen OR mobile phone OR mobile device OR tablet OR phablet OR mobile) AND
Software	(application* OR app OR service OR operating system OR android OR ios OR windows OR mobile application*) AND
Health	(health* OR medic* OR clinic* OR care OR patient) AND
mHealth	(mobile health OR mHealth OR m-Health) AND
Usability	(usab* OR understandab* OR learnab* OR operab* OR attractiv* OR user experience OR user testing OR user-centered OR ease of use) AND
Elderly	(aging OR elderly OR older adult* OR elder*)

### Selecting relevant studies to include

The inclusion criteria were smart device-based mHealth studies that (1) focused on mHealth apps, (2) conducted usability evaluations, (3) set the target users of the apps as elderly individuals, and (4) were published from January 2000 until December 2020. Only articles published in 2000 or after were selected to accommodate the release of the first touchscreen phone marketed as a smartphone [23]. The exclusion criteria were as follows: (1) non-English and non-Chinese-language publications, (2) did not specifically describe the process of usability evaluation, (3) unable to obtain full-text versions, and (4) conference abstracts. Two authors (QW and JL) independently screened the titles and abstracts first, followed by a full-text review, and conflicts were resolved through the judgment of a third author (JT) and team discussion.

### Charting data from the selected literature

The descriptive analytical method was used in this stage [35]. A data charting form was developed to guide the data extraction. The variables entered included standard bibliographical information (i.e., authors, year of publication, source of publication, country of origin), health condition/disease addressed by the app, critical measures of usability, the process of usability evaluation (methods, environment, duration, number of participants), and reflections on the evaluation methods (researchers’ discussion on evaluation methods). Full articles were imported as pdf files into NVivo software to extract, organize and search related data. Two authors (QW and JL) extracted the data independently, and the discrepancies were resolved by team consultation.

### Collating, summarizing and reporting the findings

This stage consisted of three substages: analyzing the data, reporting the results, and applying meaning to the results. For the first substage, a descriptive numerical summary was conducted to depict the characteristics and distribution of the included studies. Abductive approaches to qualitative content analysis, which combine the deductive and inductive phases, were used to analyze the data [36]. In the deductive phase, considering that the purpose of our research was to classify usability evaluation methods based on the development stages of mHealth apps and to recommend adopting a theoretical framework to systematically collate and summarize the extracted data [34], the three stages of the mHealth app usability evaluation framework were used as the theoretical categories (Table 1). The critical measures and evaluation methods of usability were classified into the appropriate theoretical categories, and the frequency of each variable was counted. In the inductive phase, the data extracted from articles and included in the variable “reflections on the evaluation methods” were read several times to summarize the statement of the advantages and disadvantages of the usability evaluation methods. Then, these statements were condensed and abstracted to interpret whether these methods were appropriate for use among elderly individuals. Finally, we identified possible gaps in the current studies and suggested evaluation methods that are suitable for elderly individuals.

## Results

### Search and screening results

The initial search obtained 1386 articles. After removing duplicates and reviewing the title, abstract and full text, 87 articles were selected. Nine more articles found through the reference list reviews were accepted. Finally, a total of 96 articles were included in this review. The flow diagram of the search procedure is presented in Fig. 1.

### Characteristics of source documents

**Fig. 2:**
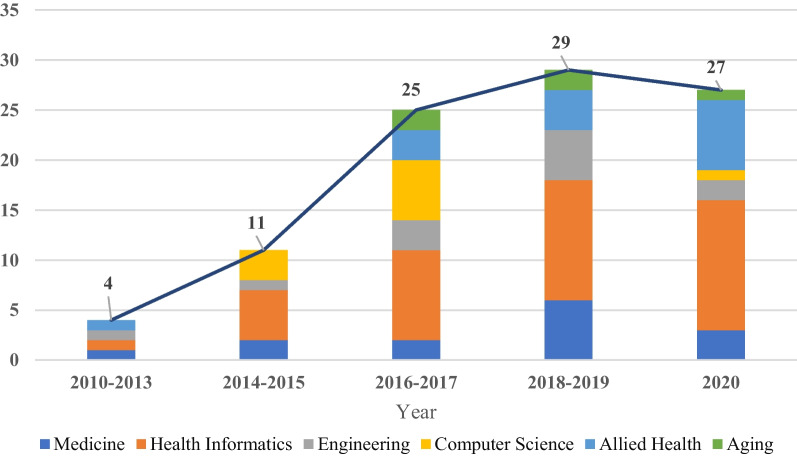
Number of articles published by year and type of journal

**Fig. 3:**
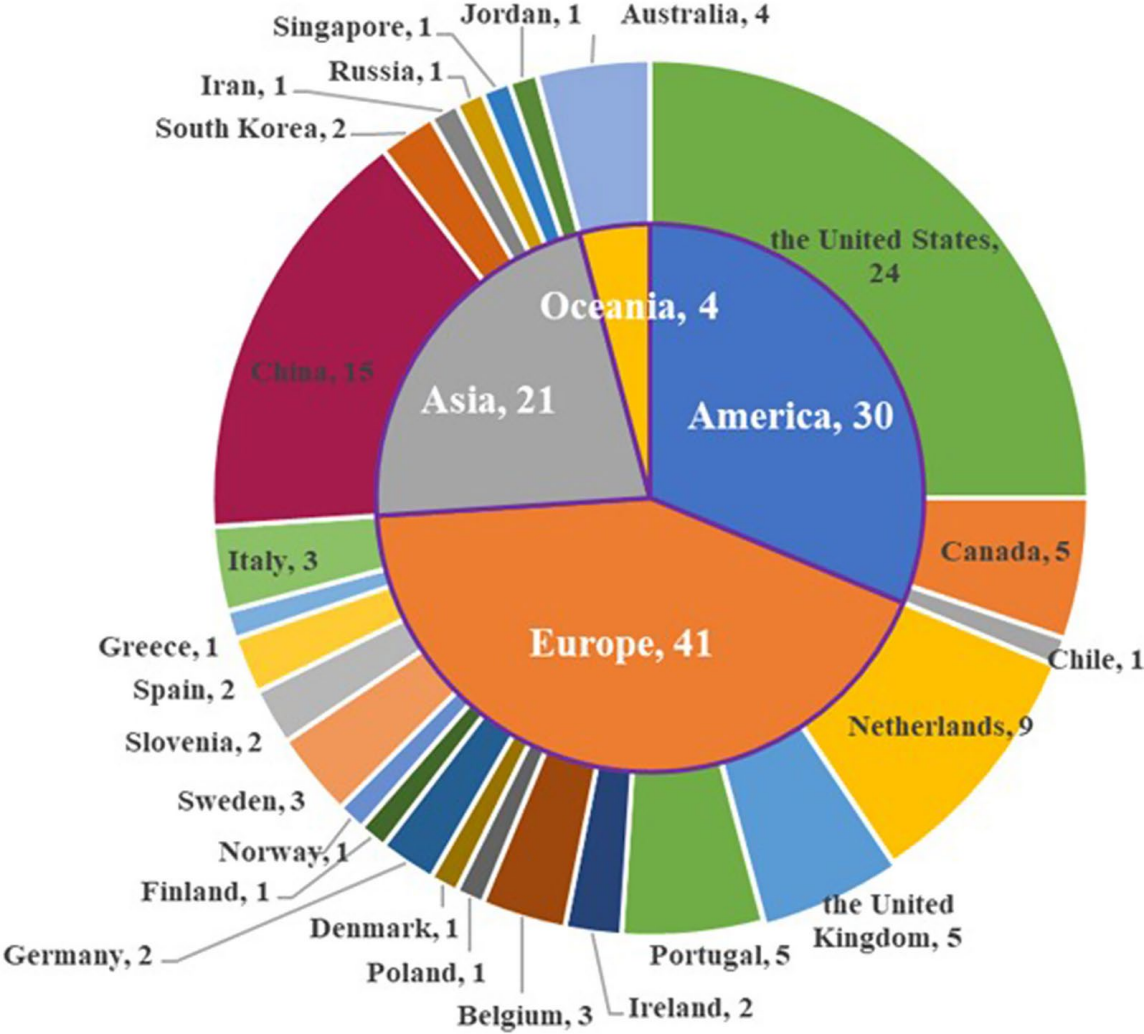
Country distribution of publications

**Fig. 4:**
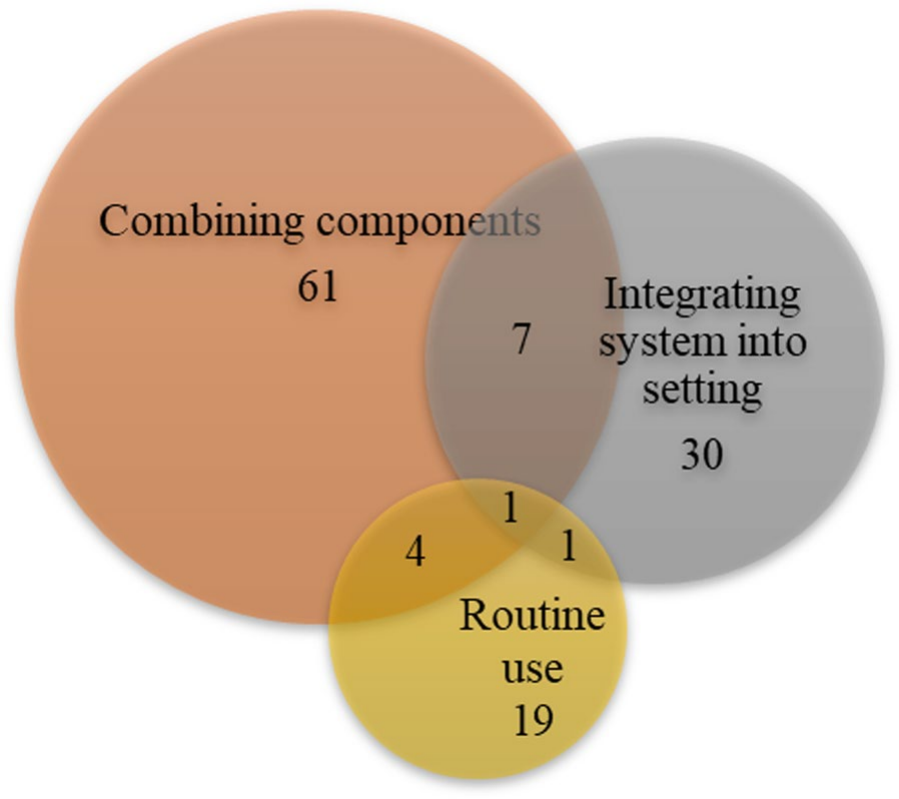
Distribution of articles under 3 stages of the mHealth app usability evaluation framework

Figure 2 shows the number of articles published per year and the types of journals. The articles were published between 2010 and 2020, with only 4 articles published before 2014 [37–40], after which the growth rate increased and peaked of 27 articles in 2020. Health informatics journals were the main publication channel, accounting for 42% (n = 40) of the selected articles. Of the 96 studies included (Fig. 3), 41 were from Europe, 30 from America, 21 from Asia, and 4 from Australia. According to the mHealth app usability evaluation framework, the distribution of articles under 3 stages is presented in Fig. 4. It is worth noting that the assessment process of 12 studies involved two stages, and one study investigated user satisfaction in different countries after diagnosing and fixing usability problems in the laboratory and real setting [2]. Slightly less than one-third (n = 29, 30.2%) of the studies reported the iterative design-evaluation process of mHealth applications by involving end users and stakeholders. Additional file : Multimedia Appendix S2 provides an overview of the articles included in the scoping review.

### Functions of the mHealth application

**Fig. 5:**
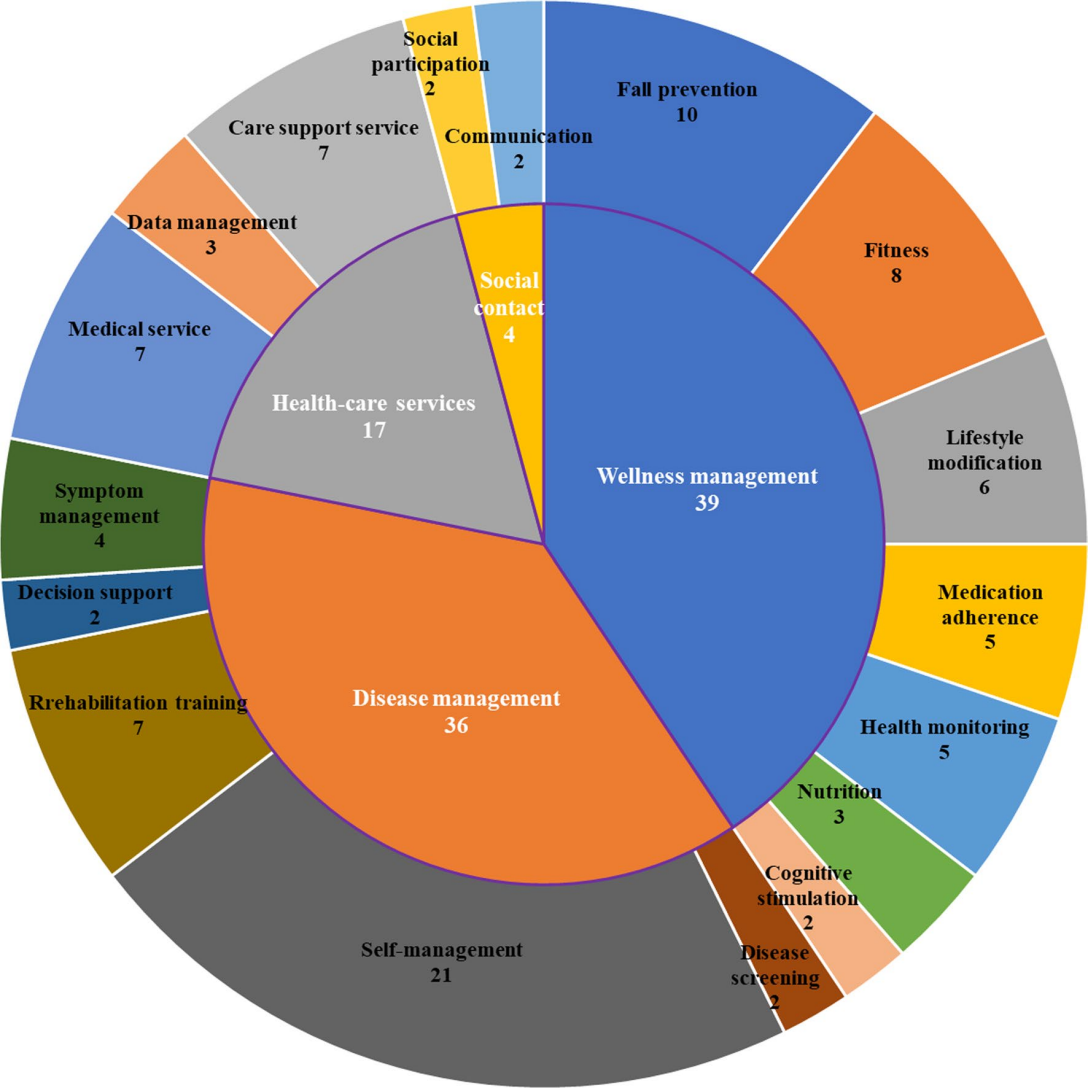
Functions of the mHealth Application

As shown in Fig. 5, the function of mHealth apps in the selected studies can be divided into four categories: wellness management (n = 39), disease management (n = 36), health-care services (n = 17), and social contact (n = 4). In the *wellness management* category, mHealth apps were used to improve the general health of older adults rather than focusing on specific diseases, which contained a variety of solutions, including fall prevention [41, 42], fitness [43, 44], lifestyle modification [45, 46], medication adherence [47, 48], health monitoring [40, 49], nutrition [50, 51], and cognitive stimulation [52, 53]. In the category of *disease management*, mHealth apps played a role in different stages of disease development, such as disease screening during diagnosis [54], decision support during treatment [33], and self-management during rehabilitation [55, 56]. In the *health-care services* category, mHealth apps have been a useful tool for helping health care providers optimize medical services and empowering users to access their health data during care transitions [38, 57, 58]. The last category of mHealth consisted of those providing *social contact*. These apps aimed to reduce social isolation and loneliness in older adults by encouraging social participation and strengthening ties with family members [59–62]. In addition, the target users of the mHealth apps in 78 (81.4%) articles were elderly individuals (aged 50/55/60/65 years or older), while others were aimed mostly at people with chronic diseases and were tested to see whether these apps were suitable for use by older people. The complete range of functions, health conditions and target users can be found in Additional file 2: Multimedia Appendix S2.

### Critical measures of usability evaluation for mHealth applications

Following the usability definitions of ISO 9241-11, ISO 25010, and Nielsen, nine critical measures of usability evaluation were extracted from the selected articles: effectiveness, efficiency, satisfaction, learnability, memorability, errors, attractiveness, operability, and understandability [14, 63, 64]. It is worth noting that effectiveness, efficiency, and satisfaction focus on the impact on users when they interact with the system, while the others concern the characteristics of the system and whether they can compensate for the decline of intrinsic capacity in elderly individuals. As shown in Table 3, the two most frequently evaluated measures are satisfaction and learnability, consistent with the dimensions of the Systems Usability Scale (SUS) [65], which was applied in 40 papers. The aspects of usability that were considered least often in the articles reviewed were errors and memorability. The assessment ratios of some critical measures were significantly related to the different stages of evaluation, indicating that the focus of the evaluation content at each stage may be different. The proportion of satisfaction and learnability in stage three was significantly higher than that in the first and second stages (*P* = 0.018 and *P* = 0.04 respectively). In contrast, the proportions of operability and comprehensibility in stages one and two were significantly higher than those in stage three (*P* = 0.02 and *P* = 0.01 respectively).

**Table 3:** Critical measures of usability evaluation for the mHealth application

Critical measures n (%)	Definition	Three stages of evaluation n (%)^a^			*P* value^b^
		1 (n = 61)	2 (n = 30)	3 (n = 19)	
Satisfaction 74 (77.1)	The extent to which the user’s physical, cognitive and emotional responses that result from the use of an app meet the user’s needs and expectations and can be expressed as interest in the app, willingness to continue using it, and initiative to share it	43 (70.5)	25 (83.3)	19 (100)	.018
Learnability 60 (62.5)	The app should be easy to learn by the class of users, which can be reflected in the introduction/instruction documents helping users to reach a reasonable level of usage proficiency within a short time	34 (55.7)	22 (73.3)	16 (84.2)	.04
Operability 50 (52.1)	The app should be easy to operate and control, which can be expressed as navigable and manipulable on the touchscreen to address the decline of cognitive ability, dexterity and muscle control in elderly individuals	36 (59.0)	19 (63.3)	5 (26.3)	.02
Understandability 40 (41.7)	The interaction information of the app should be easy to understand, which can be embodied in the clarity of the provided explanations and the graphical interface to compensate for the cognitive decline of elderly individuals	33 (54.1)	9 (30.0)	4 (21.1)	.01
Attractiveness 38 (39.6)	The interface of the app should enable pleasing and satisfying interaction for the user, for example, in terms of color use and graphic design, to meet the aesthetic needs of the elderly and accommodate their age-related perceptual resources	29 (47.4)	12 (40.0)	4 (21.1)	.12
Efficiency 33 (34.4)	The extent to which external resources, including time, human effort, money, and materials, are consumed when achieving goals by using the app	24 (39.3)	9 (30.0)	6 (31.6)	.63
Effectiveness 26 (27.1)	The extent to which actual outcomes match intended outcomes and can be measured by the accuracy and completeness with which users achieve specified goals using the app	21 (34.4)	8 (26.7)	4 (21.1)	.48
Errors 22 (22.9)	The app should have a low error rate and protect users against making errors, for example, providing error messages or help documentation to tell users how to fix problems	18 (29.5)	4 (13.3)	4 (21.1)	.22
Memorability 13 (13.5)	The operational flow of the app should be easy to remember, which can be embodied by reducing the demand on working memory through supporting recognition rather than recall	11 (18.0)	4 (13.3)	3 (15.8)	.94

^a^First stage: combining components, second stage: integrating the system into the setting, third stage: routine use

^b^Chi-squared test were conducted to reflect the statistical significance of the intergroup difference

### Empirical methods of usability evaluation for mHealth applications

Usability evaluation approaches can be classified into two categories: usability inspection and usability testing. Usability inspection is a general name for a set of methods that are all based on having experienced practitioners inspect the system using the predetermined principles with the aim of identifying usability problems [66]. In contrast, usability testing involves observing and recording the objective performance and subjective opinions of the target users when interacting with the product in order to diagnose usability issues or establish benchmarks [67].

#### Usability inspection methods

Fifteen articles used usability inspection methods to assess mHealth applications, which included two approaches: heuristic evaluation (n = 14) and cognitive walkthrough (n = 2), and one of the articles used both approaches [68].

The heuristic evaluation method requires one or more reviewers to compare the app to a list of principles that must be taken into account when designing and identifying where the app does not follow those principles [69]. In the 14 heuristic evaluation articles, the evaluators usually had different research backgrounds, such as human–computer interaction, gerontology, and specific disease areas, so that a multidisciplinary perspective could be obtained [55, 59]. The number of evaluators was in the range of 2–8, which generally referred to the suggestion by Nielsen that ‘three to five evaluators can identify 85% of the usability problems’ [63]. The heuristics can be divided into two types: generic and specific. Six studies used Nielsen’s ten principles, which are the most utilized generic heuristics [33, 40, 63]. However, traditional generic heuristics were not created for small touchscreen devices, which were the main type of app carrier, and did not consider design features that were appropriate for older adults to address their age-related functional decline in terms of perception, cognition, and movement [69]. To ensure that usability issues in these specific domains were not overlooked, the remaining eight studies extended the generic heuristics by adding usability requirements specific to elderly individuals, such as dexterity, navigation, and visual design, and finally established new heuristic checklists to evaluate the apps targeting older adults [55, 59]. Nevertheless, there was a lack of reliability analysis and expert validation for these tools except for a checklist developed by Silva [70].

Cognitive walkthrough involves one or more evaluators working through a series of tasks using the apps and describing their thought process while doing so as if they are a first-time user [71]. The focus of this method is on understanding the app’s learnability for new users [31]. The evaluators in these two studies were usability practitioners and health-care professionals [68, 72]. Before the assessment, the researchers prepared the users’ personals and the task lists [68]. During the walkthrough, the evaluators were encouraged to think aloud, and their performance was recorded by usability metrics, such as task duration and completion rate [72].

#### Usability testing methods

Almost 93% (89/96) of the studies used usability testing to evaluate mobile applications. Test participants were the target users of the apps, and they were all elderly. Some studies (n = 52) investigated the experiences of evaluators with mobile devices or their level of eHealth literacy to obtain the testing results for experts, intermediates, and novices [41, 47, 73]. The number of participants varied according to the stage and purpose of the evaluation. The average sample sizes of the first two stages were 22.8 (ranging from 2 to 189) and 15.2 (ranging from 3 to 50), respectively, with the purpose of identifying usability problems in the laboratory or real-life environment. Most of the above studies referred to Nielsen’s recommendations, which can come close to the maximum benefit–cost ratio, that is, testing three to five subjects, modifying the application, and then retesting three to five new subjects iteratively until no new major problems are identified [74]. Some studies determined the sample sizes according to the type of study design, including RCTs and qualitative research [75–77]. In stage three, usability testing was usually part of a feasibility or pilot study, and the sample size was therefore based on these design types, with an average of 60.1 (ranging from 8 to 450) [54, 78, 79].

During usability testing, the objective performance and subjective opinions of the participants were collected with the corresponding data collection methods. Thirty-four studies presented objective performance data that came from observations of operational behavior, body movements and facial expressions and could be collected by performance metrics, behavioral observation logs, screen recordings, and eye tracking [47, 72, 80, 81]. Eighty-five studies gathered the subjective opinions of the participants, which involved the users’ experience with the app and their design preferences for each part of the interface and could be investigated by means of concurrent thinking aloud, retrospective thinking aloud, questionnaires, interviews, and feedback logs [37, 41, 52, 73, 82]. The details and descriptive statistics of each data collection method are presented in Table 4.

**Table 4:** Data collection methods for usability testing

Data collection method n (%)	Description	Metrics/tools	Comments
Performance metrics 25 (26.1))	Collecting quantifiable measurements of participants’ actions during the test to understand the impacts of usability issues, usually focusing on effectiveness and efficiency	Effectiveness: number of errors, number of tasks that can be completed successfully; efficiency: task duration, number of times asking for assistance or hints, time spent recovering from errors	These quantitative indicators can be compared in young adults and seniors to reflect differences in performance [76, 81]
Behavior observation log 14 (14.6)	Observing and recording the participant’s mood and body gestures during the test	Sometimes, the observation is structured and based on predefined classifications of user behavior, such as delay or pause of > 5 s in locating the answer button [42]	This method is often used in conjunction with thinking aloud and performance metrics to improve triangulation [33]
Screen recording 3 (3.1)	Capturing the touches and actions performed on the mobile device	Screen recording software and video coding software (Behavioral Observation Research Interactive Software)	–
Eye tracking 1 (1.1)	Monitoring and recording the visual activity of the participants by tracing pupil movement within the eye	Fixations: the number of views of the area of interest; Saccade: the number of repeated visits to the specific area [52]	Because of the drooping eyelids of elderly individuals, the eye tracker may not scan their pupil accurately
Concurrent thinking aloud 25 (26.1)	Encouraging the participants to continuously verbalize their ideas, beliefs, expectations, doubts, and discoveries while performing tasks in order to understand their thoughts as they interact with the app	–	This method relies heavily on the cognitive capacities of participants, whereas these capacities decline with age; thus, it may cause reporter bias [83]
Retrospective thinking aloud 1 (1.1)	Asking the participants to view the recording of their actions and verbalize their thoughts about the tasks and the difficulties they encountered in completing the tasks	–	1. This method will increase the overall length of the evaluation and may cause the elderly to lose focus [85] 2. This method will not increase the cognitive load of the elderly compared with concurrent thinking aloud [83]
Questionnaire 68 (70.8)	Gathering the participants’ opinions about, preferences for and satisfaction with the user interface on a predefined scale after they completed the tasks	Validated questionnaires: SUS, USE, UEQ, ASQ, NASA-TLX, NPS, Health-ITUES, QUIS, PSSUQ, ICF-US, MARS, Ruland’s eight-item adaptation of Davis' ease-of-use survey, self-made questionnaires according to the unique features of a specific app	1. A larger sample size can be investigated by this method [78] 2. Some items have to be answered by an expert rather than the elderly because they are either beyond the scope of the test or based on experiencing rare occurrences. [85] 3. To prevent the response burden of the elderly and improve the understandability of the questionnaire, some items are removed or the language is modified [43, 54, 82]
Interview 36 (37.5)	Collecting the data in the form of face-to-face oral conversations with the participants, including individual interviews and focus group interviews	The interview outline: opinions on unique features, product satisfaction, and difficulties encountered during the test as well as suggestions for improvement	1. This method can obtain more new insights from the participants 2. This method is often combined with a questionnaire to collect the explanations of answers to the questionnaire
Feedback log 1 (1.1)	Asking the participants to record their experiences on a provided form when using the app	–	This method is suitable for long-term usability testing, as it can record the participant’s experience dynamically [52]

SUS, System Usability Scale [86]; USE, Usefulness Satisfaction and Ease of Use Questionnaire [87]; UEQ, User Experience Questionnaire [88]; ASQ, After Scenario Questionnaire [89]; NASA-TLX, National Aeronautics and Space Administration Task Load Index [90]; NPS, Net Promoter Score [78]; Health-ITUES, Health Information Technology Usability Evaluation Scale [91]; QUIS, Questionnaire for User Interaction Satisfaction [92]; PSSUQ, Post-Study System Usability Questionnaire [93]; ICF-US, International Classification of Functioning based Usability Scale [94]; MARS, Mobile Application Rating Scale [95]

The most frequently used collection method was questionnaires (n = 68). Of the studies, 51 used well-validated usability questionnaires, which were flexible enough to assess a wide range of technology interfaces. Frequently used usability questionnaires were the SUS (n = 40), the NPS (n = 4) and the NASA-TLX (n = 3). However, considering the lack of specificity of the standardized tool, self-designed questionnaires that lacked a reliable psychometric analysis were used in 24 studies to assess the unique features of the apps, including navigation, interface layout, and font size [45, 75, 83]. A combination of these two types of questionnaires was employed in 8 studies [59, 75, 84].

**Fig. 6:**
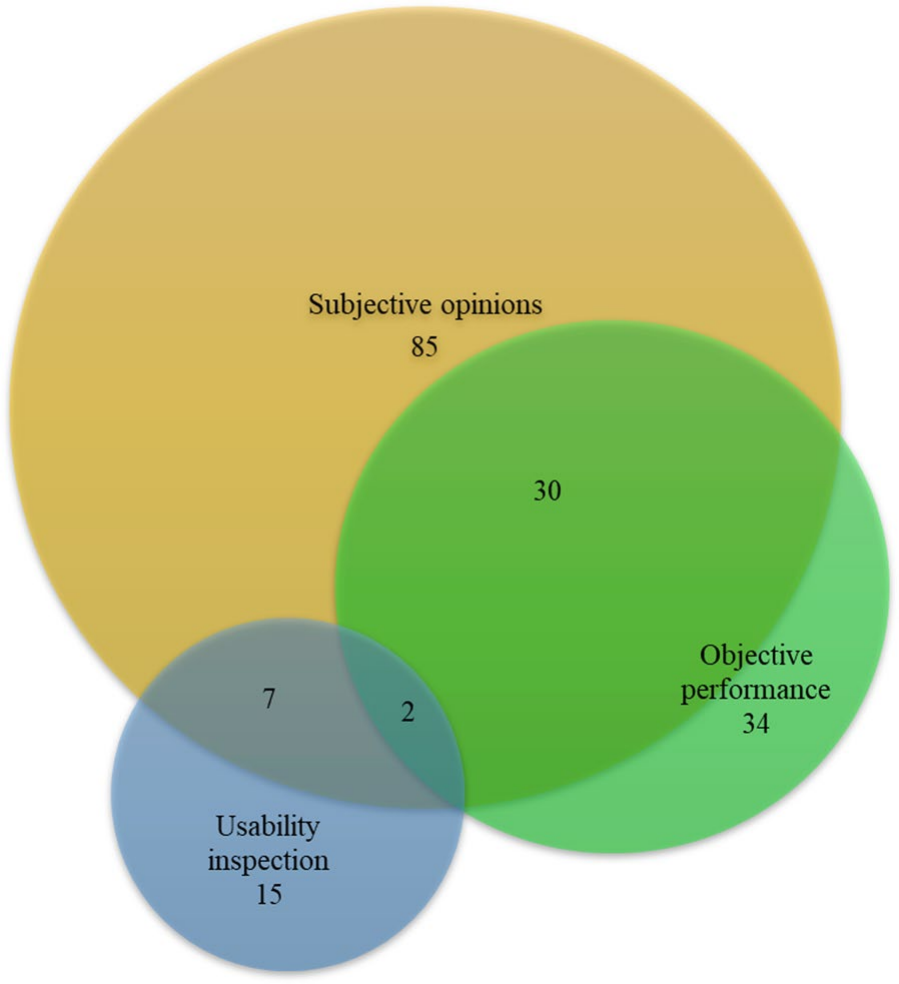
Categories of usability evaluation methods

**Fig. 7:**
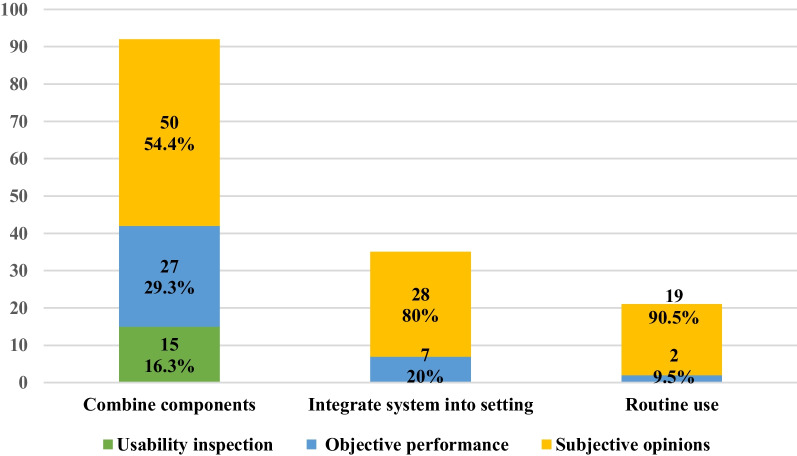
Distribution of the evaluation method types according to the mHealth app usability evaluation framework

The intersection of these methods is presented in Fig. 6. Seven studies conducted both usability inspection and usability testing. Thirty studies analyzed the results of testing based on both objective performance and subjective perceptions. Figure 7 demonstrates the distribution of the three types of evaluation methods in each stage of the mHealth app usability evaluation framework. In the three stages, most of the studies captured the subjective opinions during or after the user testing process, which was most prominent in the “routine use” stage (90.5%). The objective performance of the users was also collected at all stages, which accounted for the highest proportion in the “combining components” stage (29.3%). The usability inspection conducted by the experts was applied only in the first stage (16.3%). Table 5 illustrates the statistical description of each evaluation approach in the three stages.

**Table 5:** Usability evaluation approaches in three stages of the mHealth app usability evaluation framework

Evaluation approach	Three stages of evaluation n (%)		
	1 (n = 61)	2 (n = 30)	3 (n = 19)
Heuristic evaluation	14 (23.0)	0 (0)	0 (0)
Cognitive walkthrough	2 (3.3)	0 (0)	0 (0)
Performance metrics	19 (31.1)	5 (16.7)	2 (10.5)
Behavioral observation log	10 (16.4)	5 (16.7)	0 (0)
Screen recording	3 (4.9)	0 (0)	0 (0)
Eye tracking	1 (1.6)	0 (0)	0 (0)
Concurrent thinking aloud	22 (36.1)	4 (13.3)	0 (0)
Retrospective thinking aloud	1 (1.6)	0 (0)	0 (0)
Questionnaire	34 (55.7)	22 (73.3)	18 (94.7)
Interview	22 (36.1)	16 (53.3)	3 (15.8)
Feedback log	0 (0)	1 (3.3)	0 (0)

## Discussion

### Principal findings

This review identified 9 usability critical measures and 11 unique methods of usability evaluation and analyzed their distribution in the mHealth app usability evaluation framework. The results can assist researchers in the field of mHealth for the elderly in identifying the appropriate critical measures and choosing evaluation methods that are suitable for each usability assessment stage in the life cycle of development.

#### Emerging trends in mHealth apps to support wellness and disease management for the elderly

Overall, usability evaluation research on mHealth for the elderly has been on the rise, with a noticeable increase in 2016, and the number of articles published in 2020 was higher than that between 2010 and 2016. However, the growth rate of usability studies is far lower than the increasing number of mHealth apps. The total global mHealth market is predicted to reach nearly USD 100 billion in 2021, which would be a fivefold increase from approximately 21 billion dollars in 2016. In addition, 68% of healthcare organizations in Europe reported that they were targeting elderly people for telehealth solutions. There may be two reasons for this unequal increase. First, researchers may not realize the importance of improving the usability of mHealth apps to help the elderly overcome the digital divide [26]. Second, commercial companies developing mHealth apps are reluctant to expose usability problems to the public because of the risk of losing competitiveness [96]. In terms of app functions, wellness management and disease management have become the main types, which is consistent with recommendations for healthy aging, suggesting prevention strategies according to dynamic changes in the intrinsic abilities of the elderly [97].

#### Stage one: combining components

Approximately 64% of the studies evaluated the usability of mHealth apps at stage one, which means that most of the digital health technologies for the elderly were still in development and needed to be optimized iteratively in a controlled environment. The critical measures chosen in this phase tended to evaluate the design attributes of the system, such as understandability, operability, and attractiveness. The reason for this choice may derive from the primary purpose of this stage, which focuses on identifying usability problems rather than collecting users’ perceived ease of use or satisfaction [23, 30]. Additionally, usability inspection methods were used only in this stage. Some researchers pointed out that this type of approach should be used in the early stage of development because it is important not to expose a prototype with potential ergonomic quality control and safety problems to a vulnerable user group, such as older adults, until it has been fully inspected by experts [69, 72, 98].

#### Stage two: integrating the system into the setting

Even if a mHealth app is usable in a laboratory setting, implementation in a real environment may have different results. Therefore, stage two was carried out in realistic situations to evaluate the usability under the influence of uncontrolled environmental variables. Approximately 30% of the studies involved stage two, and eight were conducted on the basis of the optimized results in stage one. In terms of the critical measures, more research focused on the user’s subjective feelings; for example, 83.3% assessed user satisfaction in stage two and only 70.5% in stage one. The operation of the apps by the elderly was also highlighted in this stage. Age-related cognitive changes, including processing speed, executive function, and visuomotor skills, may negatively influence interactions with apps [99]. Recent design guidelines for mobile phones suggested that improving the operability of the interface, such as a simple navigation structure, could help minimize users’ cognitive load [100]. In terms of evaluation methods, most studies used questionnaires and/or interviews to collect users’ subjective opinions, and only 20% collected objective performance data. This phenomenon may be caused by the function of mHealth apps, most of which require the elderly to use them for a period of time for self-management. However, it is unrealistic and inconvenient for researchers to observe usage performance over a long period; thus, collecting perceptions after self-exploration is a viable evaluation method.

#### Stage three: routine use

After the first two stages, researchers used complete mHealth apps to conduct pilot or feasibility studies among the target population, and the usability evaluation was part of them [101]. Perceptions of satisfaction and learnability were most often evaluated, probably because almost 60% of the studies at stage three used the SUS, including two dimensions: satisfaction and learnability. In the 96 articles, there was no research to establish a usability benchmark for an app. This may be due to the large sample size required for this type of study and is usually conducted by commercial companies through market research [102].

### Gaps and potential for future research

#### The use of multiple usability evaluation methods

Several design guidelines state that a usability evaluation should include both inspection and testing methods, and inspection should be carried out before testing [31, 63, 103]. However, only two studies met the above recommendations. There are two reasons for using multiple evaluation methods. First, usability inspection methods do not have the problem of the participants in usability testing possibly not representing the pronounced heterogeneity of the target users [55]. Second, the evaluators in usability inspection are experts, thereby limiting the potential of the assessment results to provide the views of the elderly who are the end users of the app [104].

With regard to usability testing, collecting only the subjective experience of users is inadequate for identifying usability problems accurately and comprehensively [105]. However, among the 89 articles involving usability testing, 37% (n = 33) employed one evaluation method, and questionnaires were chosen in 23 of them. Specific reasons for using multiple methods to collect both subjective and objective data may be as follows. First, varying results may be obtained from different evaluation methods. One study by Richard and colleagues conducted a questionnaire survey (ASQ and NASA-TLX) from elderly users to evaluate a fall detection app [72]. The ASQ scores indicated that the users were satisfied with the product, while the NASA-TLXA and objective metrics results suggested that the app created a large mental burden for the users. The possible reason for these conflicting results was that the users judged the app to be easy and satisfactory because they completed the task successfully without considering the difficulties encountered and the time spent [72]. Second, the advantages and disadvantages of each method can supplement each other. Observational performance data collect objective behavioral characteristics of users, which cannot explain the internal mechanism of such behavior [31]. This disadvantage can be solved by analyzing user experience and preferences, which identify the cognitive process during interaction with the app [106]. Additionally, subjective opinion data are self-reported and often affected by acquiescence bias, social desirability bias, and recency bias, which leads to the underestimation of results [107]. If objective evaluation methods are also used in the test, these biases may be balanced [108]. However, using multiple evaluation methods may increase the length of testing, ultimately adding to the test burden of the elderly [33]. Thus, researchers should use the appropriate number of evaluation methods to collect subjective and objective data according to the stage of assessment, testing goals, and workload that the participants can accept.

A number of studies have pointed out that due to the decline in working memory, elderly people would frequently forget the operation steps when using mHealth apps, which is also the main reason why they give up using them [109–111]. These results all highlighted the importance of improving the memorability of the apps for elderly individuals. However, in this review, only 13 studies measured memorability, and all of them were subjectively evaluated by experts or users. One way to objectively measure memorability is to invite participants to perform a series of tasks after having become proficient in using the apps and then asking them to perform similar tasks after a period of inactivity. The two sets of results can then be compared to determine how memorable the apps were [112]. The reason for the infrequent use of this method may be the difficulty of recruiting participants who are willing to return multiple times to participate in an evaluation. Based on the above description, future research should pay attention to memorability when evaluating mHealth apps for the elderly while optimizing the objective evaluation method of this attribute to increase the recruitment rate of participants.

### Adapting usability evaluation methods to the elderly

In the context of mHealth apps for elderly individuals, it is necessary to adjust the standardized usability evaluation methods to accommodate the end users’ abilities. Standardized usability evaluation tools, such as Nielsen’s heuristics and the SUS, usually overlook specific usability issues to compensate for the decline in cognition, perception, and mobility among the elderly [98]. Thirty-two articles in this review developed their own assessment tools, of which 8 were heuristic checklists and 24 were questionnaires. However, these tools still need rigorous psychometric analysis [59].

In usability testing with older adults, researchers should choose the appropriate data collection methods according to their physiological characteristics [33]. For example, the concurrent think-aloud method requires too much attention from elderly participants with cognitive limitations, resulting in reporter bias and task execution failure [83]; thus, one study used the retrospective think-aloud method to enable the participants to explain their behavior after completing the tasks [82]. Automated usability evaluation (AUE) methods are a promising area of usability research and can improve the accuracy and efficiency of the test; thus, they may be suitable for the elderly because of the shorter timeline, preventing participants from losing focus [113, 114]. In this review, 3 papers employed an automatic capture method (screen recording and eye tracking) [72, 81, 115], and one paper used the automatic analysis method (natural language processing) [116]. In some studies, the language of the original scales is modified to match the understandability of the elderly and avoid increasing the response burden, for example, by removing a double negative from an item in the SUS or changing “cumbersome” in the SUS to “awkward” [82, 117].

The aim of researchers, designers and developers of mHealth apps should be to conduct a usability evaluation that accommodates aging barriers and possible multimorbidity issues [118]. Based on this consideration, it is necessary to choose the appropriate methods and adjust the evaluation process based on the physical function and cognitive ability of elderly users. In this review, some studies used mHealth apps to provide support activities of daily living or disease management for older adults with mental illness (dementia, cognitive impairment, schizophrenia, etc.) [117, 119–121]. Due to the limitations of the research conditions, participants in these studies were only given a short time to understand and try the apps before testing. However, such an evaluation process may not guarantee that participants fully comprehended the function of the app, given the impact of mental illness on their understanding and learning ability [122]. Meanwhile, patients with mental illness sometimes cannot express their self-feelings well [26], so using only subjective opinion report-based evaluation methods may affect the accuracy of the results. In view of the above two points, for such elderly patients, researchers should formulate appropriate app teaching programs and add objective evaluation methods to the research design.

#### Deciding the sample size of usability evaluation

Our review found that in the first two stages of the usability evaluation framework, the articles focused on detecting usability problems, and the sample size generally referred to the suggestions by Nielsen [63]. However, if the products under investigation have many problems available for discovery with probabilities of occurrence that are markedly different from the 0.31 proposed by Nielsen, then there is no guarantee that observing five participants will lead to the discovery of 85% of the problems [96]. Some researchers have suggested using complex alternative models instead of the simple binomial model to calculate the sample size [123]. However, the feasibility of such a model needs to be verified.

### Study limitations

This study may have some threats to its validity. (1) Conclusion validity: Relevant research questions may have been overlooked. Considering that this review focuses mainly on evaluation methods, the results of the usability assessment were not summarized. In future studies, the severity of usability problems in each study can be classified and rated through the user action framework (UAF) and Nielsen’s severity rating [124]. (2) Construct validity: Although the PICO criteria were used to guide the search strategies, we did not include gray literature or literature other than Chinese and English.

Given the nature of the scoping review, this study did not synthesize evidence to determine the effectiveness of usability evaluation methods. Instead, it captured the diversity of the available literature with its varied objectives, critical measures, populations, and methods. Consequently, this study was primarily exploratory and suggestive of future research directions.

## Conclusions

This scoping review provides a descriptive map of the literature on the methods used for usability evaluation of mHealth apps for elderly individuals. With the widespread popularity of mHealth applications for elderly individuals, the number of articles evaluating the usability of these techniques has grown rapidly in the past five years. mHealth apps are often used as an auxiliary means of self-management to help the elderly manage their wellness and disease. Due to the inconsistent evaluation purposes of each stage in the mHealth app usability evaluation framework, the critical measures and evaluation methods used in different stages have a certain tendency. Future research should focus on selecting specific critical measures relevant to the aging characteristics and adapting usability evaluation methods to elderly individuals by improving traditional tools, introducing automated evaluation tools and optimizing the evaluation process.

## Supplementary Information

**Additional file 1.** PRISMA-ScR-Fillable-Checklist

**Additional file 2.** 96 included articles

## Data Availability

The datasets used and/or analysed during the current study are available from the corresponding author on reasonable request.

## Abbreviations

mHealth: Mobile health
mHealth apps: Mobile health applications
SUS: Systems Usability Scale
USE: Usefulness Satisfaction and Ease of Use Questionnaire
UEQ: User Experience Questionnaire
ASQ: After Scenario Questionnaire
NASA-TLX: National Aeronautics and Space Administration Task Load Index
NPS: Net Promoter Score
Health-ITUES: Health Information Technology Usability Evaluation Scale
QUIS: Questionnaire for User Interaction Satisfaction
PSSUQ: Post-Study System Usability Questionnaire
ICF-US: International Classification of Functioning based Usability Scale
MARS: Mobile Application Rating Scale
AUE: Automated usability evaluation
UAF: User action framework
