# Supplementary material for: Usability evaluation of mHealth apps for elderly individuals: a scoping review
Source: BMC Med Inform Decis Mak. 2022 Dec 2;22:317. doi: 10.1186/s12911-022-02064-5 (PMC9717549; doi:10.1186/s12911-022-02064-5)
Supplement: Supplementary file 2 — Additional file 2. 96 included articles [file 12911_2022_2064_MOESM2_ESM.docx]

**Multimedia Appendix 2**. Description of the 96 included articles

| **No.** | **Title** | **Author** | **Year** | **Country** | **Evaluation stage** | **Functions** | **Health conditions** | **Target user** |
| --- | --- | --- | --- | --- | --- | --- | --- | --- |
| **1** | A New Application for the Motor Rehabilitation at Home: Structure and Usability of Bal-App | Elisa Pedroli | 2020 | Italy | Integrate system into setting | Disease management | Frailty | The elderly (>65) |
| **2** | Colorado Care Tablet: The design of an interoperable Personal Health Application to help older adults with multimorbidity manage their medications | Katie A. Siek | 2010 | the United States | Combine components | Disease management | Multiple chronic conditions | The elderly (>65) |
| **3** | Mobile Applications for Diabetics: A Systematic Review and Expert-Based Usability Evaluation Considering the Special Requirements of Diabetes Patients Age 50 Years or Older | Madlen Arnhold | 2014 | Germany | Combine components | Disease management | Diabetics | The elderly (>50) |
| **4** | Self-Selection of Bathroom-Assistive Technology: Development of an Electronic Decision Support System (Hygiene 2.0) | Manon Guay | 2020 | Canada | Combine components + Integrate system into setting | Health-care service | Personal hygiene | The elderly (>65) |
| **5** | Smart But Not Adapted Enough: Heuristic Evaluation of Smartphone Launchers with An Adapted Interface and Assistive Technologies for Older Adults | Andraz Petrovcic | 2017 | Slovenia | Combine components | Social contact | Suitable for aging | The elderly (>65) |
| **6** | A Software to Prevent Delirium in Hospitalised Older Adults: Development and Feasibility Assessment | Evelyn A. Alvarez | 2020 | Chile | Routine use | Health-care service | Delirium | The elderly (>65) |
| **7** | Mobile Health Technology for Atrial Fibrillation Management Integrating Decision Support, Education, and Patient Involvement: mAF App Trial | Yutao Guo | 2017 | China | Routine use | Disease management | Atrial Fibrillation | The elderly (>65) |
| **8** | Design and Usability Evaluation of Mobile Voice-Added Food Reporting for Elderly People: Randomized Controlled Trial | Ying-Chieh Liu | 2020 | China | Combine components | Wellness management | Nutrition | The elderly (>60) |
| **9** | Smartphone Apps to Support Falls Rehabilitation Exercise: App Development and Usability and Acceptability Study | Helen Hawley-Hague | 2020 | the United Kingdom | Combine components + Integrate system into setting | Wellness management | Fall prevention | The elderly (>60) |
| **10** | Supervised Digital Neuropsychological Tests for Cognitive Decline in Older Adults: Usability and Clinical Validity Study | Francesca Lunardini | 2020 | Italy | Combine components | Health-care service | Dementia and mild | The elderly (>65) |
| **11** | Mobile App Use for Insomnia Self-Management in Urban Community-Dwelling Older Korean Adults: Retrospective Intervention Study | Kyungmi Chung | 2020 | South Korea | Routine use | Disease management | Insomnia | The elderly (>60) |
| **12** | Perceived Need and Acceptability of an App to Support Activities of Daily Living in People with Cognitive Impairment and Their Carers: Pilot Survey Study | Rhoda Lai | 2020 | Australia | Combine components | Disease management | Dementia and mild cognitive | The elderly (>65) |
| **13** | Untold Stories in User-Centered Design of Mobile Health: Practical Challenges and Strategies Learned from the Design and Evaluation of an App for Older Adults with Heart Failure | Victor Philip Cornet | 2020 | the United States | Combine components | Disease management | Heart failure | The elderly (>65) |
| **14** | Using Natural Language Processing and Sentiment Analysis to Augment Traditional User-Centered Design: Development and Usability Study | Curtis Lee Petersen | 2020 | the United States | Combine components | Disease management | Sarcopenia | The elderly (>65) |
| **15** | A Mobile Phone–Based Gait Assessment App for the Elderly: Development and Evaluation | Runting Zhong | 2020 | China | Combine components | Wellness management | Physical function | The elderly (>60) |
| **16** | App-based Self-administrable Clinical Tests of Physical Function: Development and Usability Study | Ronny Bergquist | 2020 | Norway | Combine components + Integrate system into setting | Wellness management | Physical function | The elderly (>60) |
| **17** | Evaluating Mobile Health Apps for Customized Dietary Recording for Young Adults and Seniors: Randomized Controlled Trial | Ying-Chieh Liu | 2019 | China | Combine components | Wellness management | Nutrition | All crowds |
| **18** | Feasibility of Virtual Tablet-Based Group Exercise Among Older Adults in Siberia: Findings from Two Pilot Trials | Svetlana Nikitina | 2018 | Russia | Routine use | Wellness management | Exercise | The elderly (>65) |
| **19** | The Health Buddies App as a Novel Tool to Improve Adherence and Knowledge in Atrial Fibrillation Patients: A Pilot Study | Lien Desteghe | 2017 | Belgium | Integrate system into setting | Disease management | Atrial Fibrillation | The elderly (>65) |
| **20** | Human-Centered Design Study: Enhancing the Usability of a Mobile Phone App in an Integrated Falls Risk Detection System for Use by Older Adult Users | Richard Harte | 2017 | Ireland | Combine components | Wellness management | Fall prevention | The elderly (>65) |
| **21** | Cleremed: Lessons Learned from a Pilot Study of a Mobile Screening Tool to Identify and Support Adults Who Have Difficulty with Medication Labels | Kelly Anne Grindrod | 2014 | Canada | Integrate system into setting | Health-care service | Medication safety | The elderly (>55) |
| **22** | Engagement, Acceptability, Usability, and Preliminary Efficacy of a Self-Monitoring Mobile Health Intervention to Reduce Sedentary Behavior in Belgian Older Adults: Mixed Methods Study | Sofie Compernolle | 2020 | Belgium | Integrate system into setting | Wellness management | Sedentary behavior | All crowds |
| **23** | Activity Monitors as Support for Older Persons’ Physical Activity in Daily Life: Qualitative Study of the Users’ Experiences | Maria Ehn | 2018 | Sweden | Integrate system into setting | Wellness management | Exercise | All crowds |
| **24** | Study of the usability of spaced retrieval exercise using mobile devices for Alzheimer’s disease rehabilitation | Ahmad Zmily | 2014 | Jordan | Combine components | Disease management | Alzheimer's disease | The elderly (>60) |
| **25** | User-Dependent Usability and Feasibility of a Swallowing Training mHealth App for Older Adults: Mixed Methods Pilot Study | HyangHee Kim | 2020 | South Korea | Integrate system into setting | Disease management | Swallowing difficulties | The elderly (>65) |
| **26** | Implementing Mobile Health–Enabled Integrated Care for Complex Chronic Patients: Patients and Professionals’ Acceptability Study | Jordi de Batlle | 2020 | Spain | Routine use | Disease management | Chronic conditions | The elderly (>55) |
| **27** | Evaluating User Perceptions of Mobile Medication Management Applications with Older Adults: A Usability Study | Kelly Anne Grindrod | 2014 | Canada | Combine components | Wellness management | Medication adherence | Patients with chronic diseases |
| **28** | Perception of Older Adults Toward Smartwatch Technology for Assessing Pain and Related Patient-Reported Outcomes: Pilot Study | Todd Matthew Manini | 2019 | the United States | Combine components | Disease management | Chronic pain | The elderly (>65) |
| **29** | Usability of a Novel Mobile Health iPad App by Vulnerable Populations | David P Miller Jr | 2017 | the United States | Routine use | Disease management | Colorectal cancer | The elderly (>50) |
| **30** | Older Adults Can Successfully Monitor Symptoms Using an Inclusively Designed Mobile Application | Meghan Reading Turchioe | 2020 | the United States | Routine use | Disease management | Heart failure cardiovascular disease | The elderly (>65) |
| **31** | Remote Home Monitoring of Older Surgical Cancer Patients: Perspective on Study Implementation and Feasibility | Leonie T. Jonker | 2020 | Netherlands | Routine use | Health-care service | Postoperative care | The elderly (>65) |
| **32** | Does culture affect usability? A trans-European usability and user experience assessment of a falls-risk connected health system following a user-centered design methodology carried out in a single European country | Vera Stara | 2018 | Ireland | Routine use | Wellness management | Fall prevention | The elderly (>65) |
| **33** | Enabling older adults to carry out paperless falls-risk self-assessments using guidetomeasure-3D: A mixed methods study | Julian Hamm | 2019 | the United Kingdom | Combine components | Wellness management | Fall prevention | The elderly (>55) |
| **34** | Harnessing smartphone technology and three-dimensional printing to create a mobile rehabilitation system, mRehab: assessment of usability and consistency in measurement | Sutanuka Bhattacharjya | 2019 | the United States | Combine components | Disease management | Stroke | Stroke patients |
| **35** | Adapting a Psychosocial Intervention for Smartphone Delivery to Middle-aged and Older Adults with Serious Mental Illness | Karen L. Whiteman | 2017 | the United States | Combine components | Disease management | Mental illness | The elderly (>50) |
| **36** | Mobile application for diabetes self-management in China: Do they fit for older adults? | Chenchen Gao | 2017 | China | Combine components | Disease management | Diabetes | Diabetic |
| **37** | Mobile health for older adult patients: Using an aging barriers framework to classify usability problems | G.A. Wildenbos | 2019 | Netherlands | Combine components | Health-care service | Hospital appointment | The elderly (>50) |
| **38** | Usability Pitfalls of Diabetes mHealth Apps for the Elderly | Maša IsakoviT | 2016 | Slovenia | Combine components | Disease management | Diabetes | Diabetic |
| **39** | Design and evaluation of theory-informed technology to augment a wellness motivation intervention | Siobhan McMahon | 2014 | the United States | Combine components + Integrate system into setting | Wellness management | Exercise | The elderly (>60) |
| **40** | Co-Creation with Older Adults to Improve User-Experience of a Smartphone Self-Test Application to Assess Balance Function | Linda Mansson | 2020 | Sweden | Combine components | Wellness management | Fall prevention | The elderly (>60) |
| **41** | Feasibility-Usability Study of a Tablet App Adapted Specifically for Persons with Cognitive Impairment-SMART4MD (Support Monitoring and Reminder Technology for Mild Dementia) | Maria Quintana | 2020 | Spain | Combine components + Integrate system into setting | Disease management | Dementia and mild cognitive | The elderly (>50) |
| **42** | Developing an easy-to-use tablet computer application for assessing patient-reported outcomes in patients with cancer | Erik K. Fromme | 2011 | the United States | Combine components + Routine use | Health-care service | Cancer | The elderly (>60) |
| **43** | Remind Me to Remember: A pilot study of a novel smartphone reminder application for older adults with dementia and mild cognitive impairment | Katherine Hackett | 2020 | the United States | Combine components | Health-care service | Dementia and mild cognitive impairment | The elderly (>65) |
| **44** | Quality and Usability of Arthritic Pain Self-Management Apps for Older Adults: A Systematic Review | Priyanka Bhattarai | 2018 | Australia | Combine components | Disease management | Arthritic | Arthritis patients |
| **45** | User-centered development and testing of a monitoring system that provides feedback regarding physical functioning to elderly people | Joan Vermeulen | 2013 | Netherlands | Combine components + Integrate system into setting | Wellness management | Physical function | The elderly (>65) |
| **46** | "Connecting patients and therapists remotely using technology is feasible and facilitates exercise adherence after stroke" | Dawn B. Simpson | 2019 | Australia | Routine use | Disease management | Stroke | Stroke patients |
| **47** | Design and development of Medication Assistant: older adults centered design to go beyond simple medication reminders | Anto´nio Teixeira | 2017 | Portugal | Combine components | Wellness management | Medication adherence | The elderly (>60) |
| **48** | Human factors analysis, design, and evaluation of Engage, a consumer health IT application for geriatric heart failure selfcare | Preethi Srinivas | 2017 | the United States | Combine components | Disease management | Heart failure | The elderly (>65) |
| **49** | Older adults' experiences with mHealth for fall prevention exercise: usability and promotion of behavior change strategies | Marina Arkkukangas | 2020 | Sweden | Integrate system into setting | Wellness management | Fall prevention | The elderly (>65) |
| **50** | App-based attention training: Incorporating older adults' feedback to facilitate home-based use | Nikki L. Hill | 2017 | the United States | Integrate system into setting | Wellness management | cognitive abilities | The elderly (>60) |
| **51** | Empowering Senior Cochlear Implant Users at Home via a Tablet Computer Application | Birgit Philips | 2018 | Belgium | Integrate system into setting | Disease management | Disabling hearing loss | The elderly (>60) |
| **52** | Evaluation of transform Mobile eHealth Solution for Remote Patient Monitoring during Clinical Trials | JarosBaw Jankowski | 2016 | Poland | Combine components | Health-care service | Chronic conditions | Patients with chronic diseases |
| **53** | Prolonged ECG with a novel recorder utilizing electrode belt and mobile device in patients with recent embolic stroke of undetermined source: A pilot study | Tuomas Jussi Lumikari | 2020 | Finland | Routine use | Disease management | Atrial Fibrillation | The elderly (>50) |
| **54** | Ease of Use and Usefulness of Medication Reminder Apps among Rural Aging Adults | Marcia Shade | 2019 | the United States | Integrate system into setting | Wellness management | Medication adherence | The elderly (>55) |
| **55** | Development of a mobile application to screen and manage fall risks in older people | Zahra Taheri-Kharameh | 2020 | Iran | Integrate system into setting | Wellness management | Fall prevention | The elderly (>60) |
| **56** | Mobile Support for Older Adults and Their Caregivers: Dyad Usability Study | Charlene C Quinn | 2019 | the United States | Integrate system into setting | Wellness management | Elderly wellbeing | All crowds |
| **57** | A Fall Risk mHealth App for Older Adults: Development and Usability Study | Katherine L Hsieh | 2018 | the United States | Combine components | Wellness management | Fall prevention | The elderly (>65) |
| **58** | Supporting Older Adults in Exercising with a Tablet: A Usability Study | Sumit Mehra | 2019 | Netherlands | Combine components | Wellness management | Exercise | The elderly (>55) |
| **59** | Novel mHealth App to Deliver Geriatric Assessment-Driven Interventions for Older Adults with Cancer: Pilot Feasibility and Usability Study | Kah Poh Loh | 2018 | the United States | Integrate system into setting | Disease management | Cancer | The elderly (>65) |
| **60** | The Reliability of Using Tablet Technology for Screening the Health of Older Adults | Lex VAN VELSEN | 2018 | Netherlands | Combine components | Health-care service | Health screening | The elderly (>60) |
| **61** | Tablet-Based Well-Being Check for the Elderly: Development and Evaluation of Usability and Acceptability | Pradeep Ray | 2017 | China | Combine components + Integrate system into setting + Routine use | Social contact | Elderly wellbeing | The elderly (>65) |
| **62** | Increasing physical activity in older adults using STARFISH, an interactive smartphone application (app); a pilot study | Lorna Paul | 2017 | the United Kingdom | Integrate system into setting | Wellness management | Exercise | The elderly (>65) |
| **63** | A Mobile App Directory of Occupational Therapists Who Provide Home Modifications: Development and Preliminary Usability Evaluation | An Thi Nguyen | 2020 | the United States | Integrate system into setting | Health-care service | Activities of daily living | The elderly (>65) |
| **64** | The design and methodology of a usability protocol for the management of medications by families for aging older adults | Y. Quintana | 2019 | the United States | Combine components | Wellness management | Medication adherence | The elderly (>65) |
| **65** | User-Centered Evaluations with Older Adults: Testing the Usability of a Mobile Health System for Heart Failure Self-Management | Victor P. Cornet | 2017 | the United States | Combine components | Disease management | Heart failure | The elderly (>65) |
| **66** | Community-based trials of mobile solutions for the detection and management of cognitive decline | Adele Boyd | 2017 | the United Kingdom | Integrate system into setting | Wellness management | Cognitive decline | The elderly (>60) |
| **67** | Fall Prevention Self-Assessments Via Mobile 3D Visualization Technologies: Community Dwelling Older Adults' Perceptions of Opportunities and Challenges | Julian Hamm | 2017 | the United Kingdom | Combine components | Wellness management | Fall prevention | The elderly (>50) |
| **68** | Tablet-based support for older adults with severe mood disorders treated in an ambulatory geriatric psychiatry setting: Protocol of a feasibility study of the ecare@Home platform | Josien Schuurmans | 2016 | Netherlands | Integrate system into setting | Disease management | Mood disorders | The elderly (>60) |
| **69** | Acceptance factors of mobile apps for diabetes by patients aged 50 or older: a qualitative study | Madlen Scheibe | 2015 | Germany | Combine components | Disease management | Diabetes | Diabetic |
| **70** | Testing Usability and Acceptability of a Web Application to Promote Physical Activity (icanfit) Among Older Adults | Yan Hong | 2014 | the United States | Integrate system into setting + Routine use | Wellness management | Exercise | The elderly (>60) |
| **71** | The first evaluation of a Mobile application to encourage social participation for community-dwelling older adults | S. M. Jansen-Kosterink | 2020 | Netherlands | Routine use | Social contact | Social isolation | The elderly (>60) |
| **72** | A healthy lifestyle app for older adults with diabetes and hypertension: Usability assessment | Jenna Smith-Turchyn | 2017 | Canada | Combine components | Disease management | Chronic conditions | Patients with chronic diseases |
| **73** | Designing for patient-centered factors in medical adherence technology | Aisyah Hamid | 2012 | Singapore | Combine components | Wellness management | Medication adherence | The elderly (>65) |
| **74** | Evaluation of a Mobile Home Care Platform Lessons Learned and Practical Guidelines | Christos Panagopoulos | 2015 | Greece | Combine components | Wellness management | Elderly wellbeing | The elderly (>65) |
| **75** | Towards a List of Heuristics to Evaluate Smartphone Apps Targeted at Older Adults: A Study with Apps that Aim at Promoting Health and Well-being | Paula Alexandra Silva | 2015 | China | Combine components | Wellness management | Exercise | All crowds |
| **76** | Design and evaluation of a mobile user interface for older adults: navigation, interaction and visual design recommendations | Ana Correia de Barros | 2014 | Portugal | Combine components | Wellness management | Fall prevention | The elderly (>65) |
| **77** | Mobile Health Apps: Improving Usability for Older Adult Users | Stephanie A. Morey | 2019 | Australia | Combine components | Disease management | Heart failure | Heart failure patients |
| **78** | Development of a Path to Home Mobile App for the Geriatric Rehabilitation Program at Bruyere Continuing Care: Protocol for User-Centered Design and Feasibility Testing Studies | Chantal Backman | 2018 | Canada | Combine components + Routine use | Health-care service | Rahabilitation | The elderly (>65) |
| **79** | GOAL: An eHealth Application for Rewarding Healthy Behavior. The First Experiences of Older Adults | Stephanie Jansen Kosterink | 2019 | Netherlands | Combine components + Routine use | Wellness management | Elderly wellbeing | The elderly (>65) |
| **80** | Digi & Mind: Development and validation of a multi-domain digital cognitive stimulation program for older adults with cognitive decline | Filipa Couto | 2019 | Portugal | Combine components | Disease management | Cognitive abilities | The elderly (>65) |
| **81** | Engagement and Design Barriers of mHealth Applications for Older Adults | Amy Franklin | 2018 | the United States | Combine components | Wellness management | Elderly wellbeing | The elderly (>65) |
| **82** | The Gezelsch App A Dutch Mobile Application to Reduce Social Isolation and Loneliness | Stephanie Jansen - Kosterink | 2018 | Netherlands | Integrate system into setting | Social contact | Social isolation | The elderly (>60) |
| **83** | Souschef: Mobile Meal Recommender System for Older Adults | David Ribeiro | 2017 | Portugal | Combine components | Wellness management | Nutrition | The elderly (>65) |
| **84** | Usability of Mobile Consumer Applications for Individuals Aging with Multiple Sclerosis | Ljilja Ruzic | 2017 | the United States | Combine components | Wellness management | Health monitoring | All crowds |
| **85** | Universally Accessible mHealth Apps for Older Adults: Towards Increasing Adoption and Sustained Engagement | Christina N. Harrington | 2017 | the United States | Combine components | Wellness management | Elderly wellbeing | The elderly (>65) |
| **86** | Mobile Applications in the Management of Headache | Tânia Dantas | 2016 | Portugal | Combine components | Disease management | Headache | All crowds |
| **87** | Eseniorcare: Technology for Promoting Well-Being of Older Adults in Independent Living Facilities | Dipanwita Dasgupta | 2016 | Denmark | Integrate system into setting | Wellness management | Elderly wellbeing | The elderly (>60) |
| **88** | Online Group-Exercises for Older Adults of Different Physical Abilities | Marcos B´aez | 2016 | Italy | Routine use | Wellness management | Exercise | The elderly (>65) |
| **89** | Usability evaluation of mobile health App in elderly based on QFD | Jun Tang | 2018 | China | Combine components | Health-care service | Medical advice | All crowds |
| **90** | Optimal Design of APP User Experience for the Elderly Based on Conjoint Analysis | Huanhuan Liu | 2018 | China | Combine components | Health-care service | Medical advice | The elderly (>60) |
| **91** | Optimization Design Approach for User Experience of the Elderly APP Based on Kano Model and Conjoint Analysis | Yongfeng Li | 2021 | China | Combine components | Health-care service | Medical advice | The elderly (>60) |
| **92** | The development and application of a rehabilitation management mHealth APP for total knee arthroplasty based on elderly patients needs | Jinghua Xia | 2020 | China | Combine components + Routine use | Disease management | Postoperative care | The elderly (>60) |
| **93** | Design for elderly mobile medical App based on user experience | Jing Cui | 2018 | China | Integrate system into setting | Health-care service | Medical advice | The elderly (>60) |
| **94** | Study on the Construction and Evaluation of WeChat Official Account for Self-Management of Home-Based Elderly Patients with Type 2 Diabetes | Kangyao Chen | 2019 | China | Combine components + Integrate system into setting | Disease management | Diabetes | The elderly (>60) |
| **95** | The research on App interface design of mobile health care product | Jingyi Chen | 2015 | China | Integrate system into setting | Health-care service | Medical advice | The elderly (>60) |
| **96** | The design and development of a patient-centered diabetic mHealth APP and its application among empty-nest elderly patients | Xiaoyan Lv | 2019 | China | Combine components | Disease management | Diabetes | The elderly (>60) |
